# Racial Disparity in Cerebrospinal Fluid Amyloid and Tau Biomarkers and Associated Cutoffs for Mild Cognitive Impairment

**DOI:** 10.1001/jamanetworkopen.2019.17363

**Published:** 2019-12-13

**Authors:** Stephanie L. Garrett, Darius McDaniel, Malik Obideen, Antoine R. Trammell, Leslie M. Shaw, Felicia C. Goldstein, Ihab Hajjar

**Affiliations:** 1 Division of General Medicine and Geriatrics, Department of Medicine, Wesley Woods Health Center, Emory University School of Medicine, Atlanta, Georgia; 2 Department of Neurology, Emory Brain Health Center, Emory University School of Medicine, Atlanta, Georgia; 3 Division of General Medicine and Geriatrics, Department of Medicine, Emory Brain Health Center, Emory University School of Medicine, Atlanta, Georgia; 4 Department of Pathology and Laboratory Medicine, Perelman School of Medicine, University of Pennsylvania, Philadelphia; 5 Department of Neurology and Medicine, Emory Clinic at Executive Park, Emory Brain Health Center, Emory University School of Medicine, Atlanta, Georgia

## Abstract

**Importance:**

Prior evidence suggests that racial differences exist in tau biomarkers in mild cognitive impairment (MCI) and Alzheimer disease (AD). Whether this reported disparity is associated with a differential level of neurodegeneration and disease stage or with underlying mechanisms separate from amyloid or tau is unclear.

**Objectives:**

To compare cerebrospinal fluid (CSF) biomarkers in African American and white individuals with normal cognition and MCI, to estimate race-based cutoffs for these biomarkers that maximize diagnostic discrimination between normal cognition and MCI, and to study the association of demographic characteristics, cognitive performance, and common vascular risk factors with these differences.

**Design, Setting, and Participants:**

This case-control study conducted from March 1, 2016, through January 31, 2019, included participants in the Brain Stress Hypertension and Aging Research Program cohort undergoing baseline assessment. Participants were 50 years or older and recruited from the Atlanta, Georgia, area.

**Exposures:**

Self-reported race and cognitive status categorized using modified Petersen criteria and clinical consensus diagnosis.

**Main Outcomes and Measures:**

Levels of β-amyloid 1-42 (Aβ1-42), tau, and phosphorylated tau 181 (pTau181), the ratio of tau or pTau181 to Aβ1-42, and hippocampal volume on magnetic resonance imaging of the brain.

**Results:**

Data from 362 study participants were analyzed (mean [SD] age, 65.6 [7.9] years), of whom 152 (42.0%) were African American, 230 (63.5%) were women, and 189 (52.2%) had MCI. After adjustment for demographic characteristics and cognitive performance, lower mean (SE) levels were observed in African American vs white individuals with MCI for tau (52.40 [5.90] vs 78.98 [5.02] pg/mL; *P* = .001) and pTau181 (15.42 [2.06] vs 25.24 [1.75] pg/mL; *P* = .001) and a lower pTau181 to Aβ1-42 ratio (0.07 [0.02] vs 0.14 [0.01]; *P* = .003). There were no racial differences in the normal cognition group or in hippocampal volumes in the MCI group. Cutoffs for CSF biomarkers were higher for Aβ1-42 in African American relative to white individuals (208 [95% CI, 126-321] vs 197 [95% CI, 183-245] pg/mL) and lower for tau (51 [95% CI, 31-59] vs 59 [95% CI, 56-92] pg/mL) and pTau181 (12 [95% CI, 12-19] vs 20 [95% CI, 12-27] pg/mL) levels. Cutoffs for the pTau181 to Aβ1-42 ratio were 0.05 (95% CI, 0.03-0.12) for African American participants and 0.05 (95% CI, 0.05-0.13) for white participants.

**Conclusions and Relevance:**

This study found that African American individuals had lower levels of tau-based biomarkers that were not likely explained by the degree of disease stage or neurodegeneration reflected by hippocampal volumes. This study suggests that race is an important factor when interpreting CSF biomarkers, especially in the clinical diagnosis of prodromal AD. It appears that using the pTau181 to Aβ1-42 ratio may ameliorate these differences.

## Key Points

**Question:**

Given the increased risk of Alzheimer disease in African American compared with white individuals, are there underlying racial differences in clinically used cerebrospinal fluid biomarkers?

**Findings:**

In this case-control study of 362 adults 50 years or older, African American participants with mild cognitive impairment had lower biomarker levels compared with white participants with mild cognitive impairment after adjusting for demographic characteristics. African American participants also had higher levels of β-amyloid 1-42 that were not significant after adjusting for demographic characteristics and cognitive performance, and diagnostic discrimination cutoffs were higher for β-amyloid 1-42 and lower for tau biomarkers in African American participants, except for the ratio of phosphorylated tau 181 to β-amyloid 1-42.

**Meaning:**

These findings suggest that race is an important factor when interpreting cerebrospinal fluid biomarkers, and the ratio of phosphorylated tau 181 to β-amyloid 1-42 may have less racial difference than other biomarkers.

## Teaser

This case-control study compares cerebrospinal fluid (CSF) amyloid and tau biomarkers for Alzheimer disease in African American and white individuals with normal cognition and with mild cognitive impairment.

## Introduction

Identifying the early stages of Alzheimer disease (AD) is becoming an important part of the clinical assessment of mild cognitive impairment (MCI) and is standard in almost all recent clinical trials of AD prevention and therapies.^1,2,3^ This prodromal stage heavily relies on identifying evidence of AD neuropathologic changes, namely β-amyloidosis and tauopathy.^3^ The National Institute on Aging and the Alzheimer’s Association issued a biological research framework for the predementia stages of AD based on the presence of β-amyloidosis, tauopathy, and neurodegeneration.^4^ This biological framework depends on thresholds or cutoffs of the relevant biomarkers for detection of amyloidosis, tauopathy, and neurodegeneration.^4^

African American individuals are at increased risk of AD, but most AD biomarker studies have included few African American participants.^5^ Hence, whether these AD biomarkers have similar diagnostic utility in evaluating African American individuals with cognitive impairments is unclear. Emerging evidence suggests racial variability in cerebrospinal fluid (CSF) levels of tau.^6,7^ However, previous studies have not addressed whether these changes are secondary to differential disease stage or due to alternative pathways, for example, cardiovascular risk factors. Hence, whether prior observations need to be incorporated into our interpretation of CSF biomarkers in nonwhite individuals remains unclear.

Therefore, our main objective was to compare CSF β-amyloid 1-42 (Aβ1-42) and tau biomarkers (total tau and phosphorylated tau 181 [pTau181]) and their ratios as well as the level of neurodegeneration in African American vs white individuals with normal cognition or MCI. We also estimated the ability of these biomarkers to differentiate normal cognition and MCI and calculated associated cutoffs in both races that provided maximal diagnostic differentiation.

## Methods

Data for the current case-control analysis were drawn from the baseline assessment of participants in the Brain Stress Hypertension and Aging Research Program (B-SHARP) at Emory University, Atlanta, Georgia. B-SHARP participants undergo cognitive assessments, neuroimaging, and lumbar punctures and are subsequently followed up longitudinally. This analysis used data from the 362 participants enrolled from March 1, 2016, through January 31, 2019. The Emory University institutional review board approved the protocol before recruitment. Each participant provided written informed consent. This report follows the Strengthening the Reporting of Observational Studies in Epidemiology (STROBE) reporting guidelines.^8^

### Participant Description

The sample includes adults 50 years or older with normal cognition or MCI who were noninstitutionalized volunteers willing to travel to Emory University. Potential study participants were identified through a referral from the Goizueta Alzheimer’s Disease Research Center at Emory University or through strategic community partnerships with grassroots health education organizations, health fairs, advertisements, and mailed announcements. An appropriate study informant, defined as an individual who has regular contact with the participant at least once a week (in person or telephone), was also identified. Potential study participants attended a screening visit, during which they underwent cognitive testing. A study physician (A.R.T. and I.H.) performed a clinical evaluation and lumbar puncture. Participants were excluded if they had a history of stroke in the past 3 years, did not have a study informant, had a clinical diagnosis of dementia of any type, or had abnormal serum thyrotropin or vitamin B_12_ levels.

Categorization of MCI used the modified Petersen criteria. This modification included using the Montreal Cognitive Assessment (MoCA)^9^ instead of the Mini-Mental State Examination.^10^ Criteria for MCI included subjective memory concerns, an MoCA score of less than 26, Clinical Dementia Rating Memory Sum of Boxes score of 0.5,^11^ educational level–adjusted cutoff score on Logical Memory Delayed Recall of the Wechsler Memory Scale,^12^ and preserved instrumental activities of daily living (Functional Assessment Questionnaire) score of 7 or less.^13^ Normal cognition was defined as having no significant memory concerns beyond those expected for age, a MoCA score of at least 26 points, a Clinical Dementia Rating score of 0 (including a 0 on the Memory Sum of Boxes score), and preserved Functional Assessment Questionnaire score of 7 or less. Each participant’s evaluation results underwent a review with the study physician and principal investigator (I.H.) and the study neuropsychologist (F.C.G.). In instances when assessment did not reveal a clear cognitive category (eg, one measure was inconsistent with the other cutoffs for a group classification, or there was a conflicting informant or participant report), a consensus diagnosis was sought by reviewing the physician interview and other relevant elements (F.C.G. and I.H.).^14^ If the 2 evaluators failed to reach an agreement, then a third independent cognitive neurologist from Emory University who was blinded regarding the initial evaluation diagnosis was consulted for a tiebreaker assessment.

### Assessment and Biomarker Measurements

Demographic characteristics (age, sex, race, and educational attainment), anthropometric characteristics (weight and height), medical diagnosis, and income levels were collected at baseline by interview. After a fast of no less than 6 hours, CSF samples were collected via lumbar puncture using 24G atraumatic spinal needles (Sprotte; Teleflex Medical). Samples were collected in sterile polypropylene tubes, separated into 0.5-mL aliquots, and stored at −80 °C. Samples were subsequently shipped to and analyzed by the Biomarker Research Laboratory at the University of Pennsylvania, Philadelphia (to L.M.S.).^15^ Levels of CSF biomarkers Aβ1-42, tau, and pTau181 were measured using a multiplex platform (xMAP; Luminex Corp) with immunoassay kit–based reagents (INNO-BIA AlzBio3; Innogenetics; for research use–only reagents). The test-retest reliabilities are 0.98 for tau, 0.90 for Aβ1-42, and 0.85 for pTau181. Cerebrospinal fluid levels of Aβ1-42 and tau are stable during repeated testing (coefficients of variation, 4.4%-6.1%).

### Magnetic Resonance Imaging of the Brain

Magnetic resonance imaging (MRI) of the brain was also completed at Emory University (3.0-T Trio MRI scanner; Siemens Medical Solutions). High-resolution T1-weighted images were acquired using a magnetization-prepared rapid gradient-echo imaging sequence with field of view of 256 × 256 mm^2^; 176 sagittal slices; isotropic voxel resolution, 1.0 mm^3^; repetition time, 2300 milliseconds; echo time, 2.89 milliseconds; inversion time, 800 milliseconds; flip angle, 8°; and scan duration, 8 minutes 37 seconds. Quality checks included head motion detection and/or correction, atlas registration confirmation, and visual inspection of images.

Hippocampal volume and other volumetric measurements were calculated using the FreeSurfer package, version 6.0.0, with manual supervision. Quality checks were performed for each scan. Left and right hippocampal volumes were obtained and combined to derive the total hippocampal volume. Intracranial volume was also derived from this analysis. Volumetric measurements using FreeSurfer have been shown to provide similar estimates to a fully manual procedure.^16^ We used intracranial volume–adjusted hippocampal volume to reflect the degree of neurodegeneration for each participant.^4^

### Statistical Analysis

Study participant characteristics were compared between the 2 racial groups (African American vs white) and cognitive groups (MCI vs normal cognition) using analysis of variance or χ^2^ statistics and presented as means with SDs and counts with percentages. Race was identified by study participants’ self-report; no genetic analysis was incorporated in the present study. Cerebrospinal fluid biomarkers (Aβ1-42, tau, pTau181, tau to Aβ1-42 ratio, and pTau181 to Aβ1-42 ratio) did not deviate from normality and were used as continuous outcome variables in all analyses. Biomarker comparisons between the 2 racial groups were performed using general linear models.

Covariate adjustments were performed on potential factors that may affect or confound racial disparity in AD risk and cognitive performance, including age, sex, educational level, body mass index (BMI; calculated as weight in kilograms divided by square of height in meters), and family history of AD. We included MoCA scores in the multivariate models to potentially account for the difference in cognitive reserve by race^17^ and account for the possibility that biomarker differences are a result of the different stages of disease within the cognitively impaired group. Hippocampal volume comparisons were further adjusted for the estimated intracranial volume. An interaction factor (race × MCI) was included in the models.

We then conducted receiver operating characteristic (ROC) curve analyses to assess the performance of each biomarker in differentiating normal cognition vs MCI by race. We generated an area under the curve (AUC) for MCI in African American and white individuals for each biomarker (considered separately) and the 2 ratios. We also calculated cutoff values for CSF biomarkers using the Youden J index (sensitivity plus [specificity − 1]) in ROC curve analysis to identify the cutoff point that optimizes the biomarker’s differentiating ability when equal weight is given to sensitivity and specificity.^18^ We calculated 95% CIs around the univariate estimate of the cutoff using bootstrap with resampling ×1000.^19^ We also obtained covariate-adjusted cutoffs to study their association with the cutoffs (the latter are presented in the eTable in the Supplement). All statistical analyses were performed using SAS, version 9.4 (SAS Institute Inc), and R, version 3.5.1 (R Project for Statistical Computing). Two-sided *P* < .05 indicated significance.

## Results

### Sample Description

Data from 362 study participants (mean [SD] age, 65.6 [7.9] years; 152 African American [42.0%]; 230 women [63.5%] and 132 men [36.5%]; 189 with MCI [52.2%]) in the B-SHARP cohort were included in the analysis. The description of the study sample is highlighted in Table 1. African American participants were younger (mean [SD] age, 63.2 [6.9] vs 67.4 [8.2] years; *P* < .001), had fewer years of formal education (mean [SD], 15.0 [2.5] vs 16.3 [2.8] years; *P* < .001), had higher BMI (mean [SD], 30.4 [6.6] vs 26.5 [5.5]; *P* < .001), and were nearly 2 times as likely to have hypertension (101 [69.2%] vs 76 [37.3%]; *P* < .001). White participants had a higher prevalence of a family history of AD (63 [41.7%] vs 125 [61.0%]; *P* = .001). The prevalence of MCI was not statistically different between the 2 groups, yet MoCA scores were lower in the African American participants (mean [SD], 23.0 [3.6] vs 24.4 [3.8]; *P* = .001).

**Table 1.** Comparisons of Key Characteristics in the Normal Cognitive and MCI Groups of White and African American Study Participants^a^

Characteristic	Diagnostic Group				*P* Value
	Normal Cognition		MCI		
	White (n = 106)	African American (n = 67)	White (n = 104)	African American (n = 85)	
Age, mean (SD), y	64.7 (7.5)	62.8 (6.1)	70.1 (8.1)	63.4 (7.4)	<.001
Sex					
Female	70 (66.0)	47 (70.1)	61 (58.7)	52 (61.2)	.42
Male	36 (34.0)	20 (29.9)	43 (41.3)	33 (38.8)	
Education					
Some high school	1 (0.9)	0	2 (1.9)	3 (3.5)	<.001
High school diploma or GED	4 (3.8)	6 (9.0)	13 (12.5)	19 (22.4)	
Associate degree, some college, or vocational school	20 (18.9)	26 (38.8)	18 (17.3)	31 (36.5)	
Bachelor or college degree	34 (32.1)	16 (23.9)	33 (31.7)	16 (18.8)	
Postgraduate study	47 (44.3)	19 (28.4)	38 (36.5)	16 (18.8)	
BMI^b^					
Underweight	1 (0.9)	2 (3.2)	5 (4.9)	0	<.001
Normal or healthy weight	48 (45.3)	10 (15.9)	44 (43.1)	18 (21.2)	
Overweight	32 (30.2)	23 (36.5)	28 (27.5)	29 (34.1)	
Obese	20 (18.9)	21 (33.3)	22 (21.6)	31 (36.5)	
Morbid obesity	5 (4.7)	7 (11.1)	3 (2.9)	7 (8.2)	
CDR Memory Sum of Boxes score^c^					
0	103 (98.1)	65 (97.0)	9 (9.0)	12 (14.1)	<.001
0.5	2 (1.9)	2 (3.0)	91 (91.0)	73 (85.9)	
MoCA score^d^					
≥26	86 (81.1)	34 (50.7)	7 (6.8)	5 (5.9)	<.001
<26	20 (18.9)	33 (49.3)	96 (93.2)	80 (94.1)	
Family history of AD	62 (60.2)	31 (47.0)	63 (61.8)	32 (37.6)	.002
Hypertension	51 (48.6)	48 (75.0)	25 (25.3)	53 (64.6)	<.001
Diabetes	8 (7.6)	16 (25.0)	7 (7.0)	18 (22.0)	<.001
Heart disease	16 (15.1)	18 (26.9)	26 (25.0)	19 (22.4)	.22
High cholesterol level	51 (49.0)	31 (48.4)	38 (38.8)	39 (47.6)	.45
Stroke	3 (2.9)	2 (3.1)	2 (2.0)	2 (2.4)	.97
Hippocampal volume, mean (SD), mm^3^	7615 (902)	7344 (814)	6738 (1218)	7258 (826)	<.001

Abbreviations: AD, Alzheimer disease; BMI, body mass index; CDR, Clinical Dementia Rating; GED, General Educational Development; MCI, mild cognitive impairment; MoCA, Montreal Cognitive Assessment.

^a^ Data are missing for some participants for BMI, CRD, family history of AD, hypertension, diabetes, high cholesterol level, and stroke.

^b^ Normal or healthy weight indicates BMI (calculated as weight in kilograms divided by height in meters squared) of 18.5 to 24.9; overweight, 25.0 to 29.9; and obesity, greater than or equal to 30.0.

^c^ Higher scores indicate worse cognition.

^d^ Higher scores indicate better cognition.

### Biomarkers and Hippocampal Volume by Race

The means (SDs) of the concentrations of AD biomarkers are shown in Table 2. We observed racial differences in all CSF biomarkers in the MCI group before covariate adjustments. These differences remained significant after covariate adjustment except for Aβ1-42 level. After adjusting for covariates, African American participants with MCI had nonsignificant higher mean concentration of Aβ1-42 compared with white participants with MCI (difference, 21.24 pg/mL; 95% CI, −10.64 to 53.12 pg/mL). Conversely, African American compared with white participants with MCI had significantly lower concentrations of total tau (difference, −26.58 pg/mL; 95% CI, −42.75 to −10.41 pg/mL) and pTau181 (difference, −9.82 pg/mL; 95% CI, −15.47 to −4.17 pg/mL). The tau to Aβ1-42 ratio (difference, −0.19; 95% CI, −0.34 to −0.04) and the pTau181 to Aβ1-42 ratio (difference, −0.06; 95% CI, −0.11 to −0.02) were lower for African American compared with white participants in the MCI group. There was no difference between participants with normal cognition.

**Table 2.** Unadjusted Mean and Covariate-Adjusted LSM Concentrations of 3 CSF Biomarkers and 2 Ratios by Race in B-SHARP Cohort

Biomarker by Study Population	Unadjusted Analysis			Adjusted Analysis			Difference (95% CI)^c^
	Racial Group, Mean (SE)		*P* Value	Racial Group, LSM (SE)^a^		*P* Value^b^	
	African American	White		African American	White		
Aβ1-42 level, pg/mL							
Full	247.76 (77.79)	235.33 (74.50)	.16	249.35 (8.27)	239.09 (6.34)	.35	10.26 (−11.18 to 31.71)
MCI	246.68 (81.50)	206.40 (73.52)	.001	246.01 (11.64)	224.77 (9.90)	.19	21.24 (−10.64 to 53.12)
Normal cognition	249.18 (73.39)	261.42 (65.47)	.30	254.18 (11.38)	256.54 (7.73)	.87	−2.36 (−30.53 to 25.82)
Tau level, pg/mL							
Full	48.00 (29.51)	69.80 (45.52)	<.001	48.15 (3.89)	65.16 (2.98)	.001	−17.01 (−27.10 to −6.92)
MCI	50.59 (29.26)	86.55 (56.39)	<.001	52.40 (5.90)	78.98 (5.02)	.001	−26.58 (−42.75 to −10.41)
Normal cognition	44.58 (29.79)	54.70 (24.68)	.03	48.03 (4.49)	50.93 (3.05)	.61	−2.9 (−14.02 to 8.22)
pTau181 level, pg/mL							
Full	13.25 (6.37)	20.31 (14.82)	<.001	13.60 (1.28)	19.81 (0.98)	<.001	−6.21 (−9.54 to −2.88)
MCI	13.83 (6.72)	25.92 (18.19)	<.001	15.42 (2.06)	25.24 (1.75)	.001	−9.82 (−15.47 to −4.17)
Normal cognition	12.49 (5.86)	15.24 (8.17)	.04	13.08 (1.33)	14.23 (0.90)	.49	−1.15 (−4.44 to 2.15)
Tau to Aβ1-42 ratio							
Full	0.23 (0.27)	0.36 (0.38)	.001	0.22 (0.03)	0.33 (0.03)	.009	−0.12 (−0.20 to −0.03)
MCI	0.25 (0.34)	0.51 (0.48)	<.001	0.25 (0.06)	0.44 (0.05)	.01	−0.19 (−0.34 to −0.04)
Normal cognition	0.19 (0.11)	0.23 (0.16)	.09	0.21 (0.03)	0.22 (0.02)	.75	−0.01 (−0.07 to 0.05)
pTau181 to Aβ1-42 ratio							
Full	0.06 (0.06)	0.11 (0.11)	<.001	0.06 (0.01)	0.10 (0.01)	.001	−0.04 (−0.06 to −0.02)
MCI	0.07 (0.08)	0.15 (0.13)	<.001	0.07 (0.02)	0.14 (0.01)	.003	−0.06 (−0.11 to −0.02)
Normal cognition	0.05 (0.03)	0.06 (0.04)	.12	0.06 (0.01)	0.06 (0.01)	.60	0.00 (−0.02 to 0.01)

Abbreviations: Aβ1-42, β-amyloid 1-42; B-SHARP, Brain Stress Hypertension and Aging Research Program; LSM, least square means; MCI, mild cognitive impairment; pTau181, phosphorylated tau 181.

^a^ Derived from the general linear models after adjustment for age, sex, educational level, family history of Alzheimer disease, body mass index, Montreal Cognitive Assessment score, hypertension, diabetes, and income level.

^b^ Bonferroni correction (*P* < .017) did not alter the results for the adjusted *P* values.

^c^ Derived from the general linear model using the SAS code statement for the difference between least square means with associated 95% CI.

**Figure 1.**
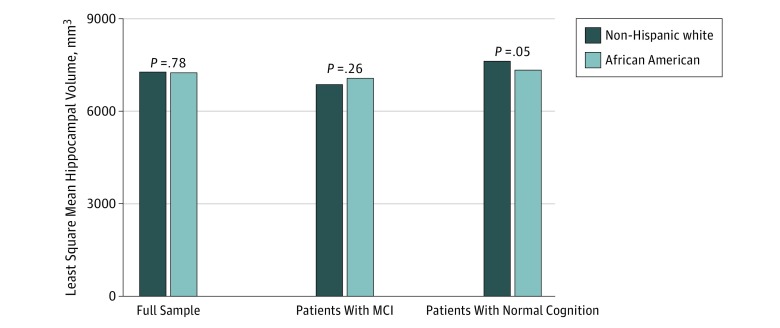
Least Square Means of the Hippocampal Volume Data are stratified by race for the full sample and in those with mild cognitive impairment (MCI) and normal cognition. Values are adjusted for age, sex, educational level, body mass index, family history of Alzheimer disease, Montreal Cognitive Assessment scores, and intracranial volume.

Hippocampal volume, adjusted for intracranial volume and other covariates, was not different between African American and white participants in the full sample (7244.9 vs 7276.2 mm^3^; *P* = .78). It was numerically but not statistically significantly higher in African American compared white participants with MCI (7062.2 vs 6876.1 mm^3^; *P* = .26) and lower in African American compared with white participants with normal cognition (7341.2 vs 7628.3 mm^3^; *P* = .05). Testing for an interaction effect between race and cognitive category further revealed no significance (*P* = .11). These results are shown in Figure 1.

### ROC Analysis and Estimated Cutoffs

**Figure 2.**
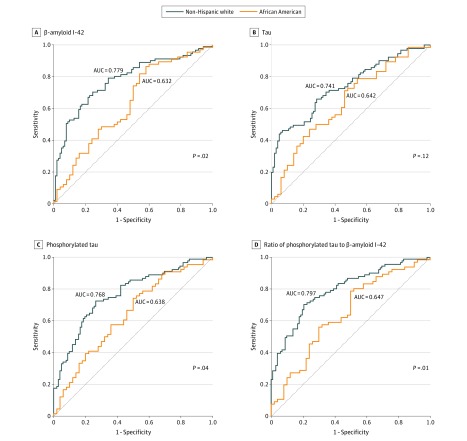
Receiver Operating Characteristic Curve Analysis With Areas Under the Curve (AUC) Data are given for cerebrospinal fluid biomarkers in white and African American participants.

Receiver operating characteristic analysis consistently showed lower AUC for all CSF biomarkers in African American compared with white participants. This was significant for Aβ1-42 level (AUC, 0.632 vs 0.779; *P* = .02), pTau181 level (AUC, 0.638 vs 0.768; *P* = .03), and pTau181 to Aβ1-42 ratio (AUC, 0.647 vs 0.797; *P* = .01) but not for tau level or tau to Aβ1-42 ratio. These results are shown in Figure 2. We then estimated the cutoffs for each biomarker for maximal discrimination between normal cognition and MCI. These calculations are shown in Table 3. Before accounting for demographic characteristics, educational level, family history of AD, BMI, and cognitive scores, the MCI cutoff for Aβ1-42 level was higher (208 [95% CI, 126-321] vs 197 [95% CI, 183-245] pg/mL), and the cutoffs for tau level (51 [95% CI, 31-59] vs 59 [95% CI, 56-92] pg/mL), pTau181 level (12 [95% CI, 12-19] vs 20 [95% CI, 12-27] pg/mL), and tau to Aβ1-42 ratio (0.17 [95% CI, 0.13-0.46] vs 0.23 [95% CI, 0.22-0.33]) were lower in African American compared with white participants. After adjusting for these factors, the differences by race for Aβ1-42 (265 vs 193 pg/mL) and tau (71 vs 28 pg/mL) levels increased (eTable in the Supplement). Of all the measures, the pTau181 to Aβ1-42 ratio showed the least racial difference in the estimated cutoffs (African American participants, 0.05 [95% CI, 0.03-0.12]; white participants, 0.05 [95% CI, 0.05-0.13]). Across all measures, specificity and sensitivity for the biomarkers were higher in white compared with African American participants. Covariate adjustment lowered these racial differences but did not account for the differences (eTable in the Supplement).

**Table 3.** Cerebrospinal Fluid Cutoffs for Maximal Discrimination Between Normal Cognition and Mild Cognitive Impairment by Race Using Receiver Operating Characteristic Curve Analyses

Race by CSF Biomarker	Cutoff (95% CI)^a^	Sensitivity	Specificity	PPV	NPV
African American					
Aβ1-42 level, pg/mL	208 (126-321)	0.39	0.74	0.67	0.48
Tau level, pg/mL	51 (31-59)	0.39	0.82	0.74	0.51
Tau to Aβ1-42 ratio	0.17 (0.13-0.46)	0.5	0.66	0.66	0.5
pTau181 level, pg/mL	12 (12-19)	0.62	0.62	0.68	0.55
pTau181 to Aβ1-42 ratio	0.05 (0.03-0.12)	0.44	0.70	0.66	0.49
White					
Aβ1-42 level, pg/mL	197 (183-245)	0.55	0.85	0.77	0.68
Tau level, pg/mL	59 (56-92)	0.66	0.72	0.68	0.7
Tau to Aβ1-42 ratio	0.23 (0.22-0.33)	0.74	0.72	0.70	0.75
pTau181 level, pg/mL	20 (12-27)	0.57	0.78	0.69	0.66
pTau181 to Aβ1-42 ratio	0.05 (0.05-0.13)	0.78	0.65	0.67	0.77

Abbreviations: Aβ1-42, β-amyloid 1-42; CSF, cerebrospinal fluid; NPV, negative predictive value; PPV, positive predictive value; pTau181, phosphorylated tau 181.

^a^ Calculated using logistic regression with receiver operating characteristic curve analysis to maximize Youden J index (the largest vertical distance from the uninformative diagonal line). The 95% CIs are calculated using bootstrap with sampling (×1000). Covariate-adjusted cutoffs are provided in eTable in the Supplement.

## Discussion

This study indicates that African American participants had lower tau-based biomarker levels that were not accounted for by differences in cognitive performance, level of neurodegeneration, or vascular risk factors. Our results provide additional support to the prior reports of lower tau measures in African American participants^6,7^ but extend these observations to show that they do not reflect differences in disease stage, because the findings were independent of overall cognitive scores and hippocampal atrophy. Alternative mechanisms are likely involved in these differences.

One such explanation would be that the cognitive impairment and associated lower tau and pTau181 levels seen in African American individuals are associated with the higher cardiovascular comorbidities and risk factors seen in this population. However, when we adjusted for cardiovascular risk factors such as BMI, type 2 diabetes, and hypertension, none of these factors explained the racial disparity. Research into other vascular or cerebrovascular factors may offer further insight into explaining these disparities. Previous racial neuropathological comparisons suggest more mixed pathologic findings in postmortem African American brains with clinical diagnosis of AD, including Lewy bodies, atherosclerosis, and arteriosclerosis.^20^ These factors may offer an additional explanation beyond the currently measured factors considered in this report.

In contrast to prior studies, we observed higher levels of Aβ1-42 in the African American group. However, these differences were not significant after adjusting for demographic characteristics, BMI, and cognitive scores. Nevertheless, although these differences did not meet the threshold of statistical significance, they may reflect an underlying clinical importance, because current diagnostic recommendations use unadjusted levels. Our analysis of cutoff by race supports this observation that consideration of demographic characteristics and race in defining diagnostic categories, including those associated with Aβ1-42 levels, are paramount.

We saw a tendency for higher hippocampal volume in the African American participants with MCI and a lower volume in African American participants with normal cognition. Although these differences did not reach statistical significance, they may show a trend for a lower reserve, with more cognitive decline in the setting for a higher hippocampal volume. Future studies with longitudinal measures of brain structure may offer insights underlying racial disparities in hippocampal sizes and other factors associated with cognitive reserve.^17^

Overall, diagnostic performance of CSF biomarkers was universally lower among African American compared with white participants. Accounting for demographic characteristics and other factors lowered these disparities but did not totally account for them. These observations highlight the importance of interpreting these measures with demographically appropriate cutoffs. Such consideration has been widely applied to the interpretation of cognitive test scores and likely is needed for AD biomarkers. These observations also highlight the importance of accounting for the complexities of health in diverse populations when using biomarkers as recommended in the National Institute on Aging–Alzheimer’s Association research framework.^4^

Recent studies have suggested that the Aβ1-42 to pTau181 ratio may have the greatest potential for diagnostic accuracy in AD.^21^ Our study suggests that diagnostic performance of the ratio cutoff is less influenced by race. The clinical implication is that relying on this ratio may lower the association of demographic characteristics and race with the CSF biomarkers, hence minimizing overdiagnosis or underdiagnosis of prodromal AD.

Our observation that race has a significant association with biomarker levels and their diagnosis discrimination performance has important public health implications. Increasingly, patients and research participants are being evaluated, recruited, and exposed to treatments or investigative agents based on their biomarker levels. Ignoring these racial differences and the effect of demographical adjustments may affect the accuracy of the diagnosis and the outcome of clinical trials.

### Strengths and Limitations

The strengths of our report include the relatively larger number of African American participants with MCI represented in the sample, the investigation of diagnostic performances of the most commonly used biomarkers in clinical practice, and the exploration of potential factors accounting for these racial factors. However, the absence of pathologic confirmation of the underlying AD changes and the cross-sectional nature of the study need to be considered when interpreting these results. Another limitation is that these results cannot be generalized beyond the self-selected individuals who agreed to participate in this study and undergo CSF analysis.

## Conclusions

The results of the present study suggest that for a comparable level of cognitive impairment and hippocampal volume, African American individuals with MCI have lower levels of tau and pTau181 and lower tau to Aβ1-42 and pTau181 to Aβ1-42 ratios. Associated cutoff points and diagnostic performance were also associated with race and may be improved by accounting for demographic characteristics. The pTau181 to Aβ1-42 ratio may be the least affected by racial differences. These findings suggest that clinical and research criteria for prodromal AD need to be interpreted in the context of these racial disparities.

## Notes

Supplement. **eTable.** CSF Cutoffs for Maximal Discrimination Between Normal Cognition vs MCI by Race With Adjustment to Relevant Covariates
