# Supplementary material for: Racial Disparity in Cerebrospinal Fluid Amyloid and Tau Biomarkers and Associated Cutoffs for Mild Cognitive Impairment
Source: JAMA Netw Open. 2019 Dec 13;2(12):e1917363. doi: 10.1001/jamanetworkopen.2019.17363 (PMC6991300; doi:10.1001/jamanetworkopen.2019.17363)
Supplement: Supplement. — eTable. CSF Cutoffs for Maximal Discrimination Between Normal Cognition vs MCI by Race With Adjustment to Relevant Covariates [file jamanetwopen-2-e1917363-s001.pdf]

Supplementary Online Content

Garrett SL, McDaniel D, Obideen M, et al. Racial disparity in cerebrospinal fluid amyloid and tau biomarkers and associated cutoffs for mild cognitive impairment. *JAMA Netw Open.* 2019;2(12):e1917363. doi:10.1001/jamanetworkopen.2019.17363

eTable. CSF Cutoffs for Maximal Discrimination Between Normal Cognition vs MCI by

Race With Adjustment to Relevant Covariates

This supplementary material has been provided by the authors to give readers additional information about their work.

© 2019 Garrett SL et al. *JAMA Network Open*

eTable. CSF Cutoffs for Maximal Discrimination Between Normal Cognition vs MCI by Race With Adjustment to Relevant Covariates

		**Adjusted**				
Race	CSF	**Cutoff**	Sensitivity	Specificity	PPV	NPV
African American	Aβ1-42	**265**	0.88	0.65	0.77	0.8
	Tau	**71**	0.77	0.77	0.82	0.71
	Tau/ Aβ1-42	**0.13**	0.79	0.77	0.83	0.73
	Ptau181	**14**	0.91	0.63	0.77	0.83
	Ptau181/ Aβ1-42	**0.04**	0.7	0.83	0.85	0.67
White	Aβ1-42	**193**	0.96	0.78	0.8	0.95
	Tau	**28**	0.79	0.94	0.92	0.83
	Tau/ Aβ1-42	**0.12**	0.82	0.91	0.89	0.85
	Ptau181	**13**	0.9	0.84	0.84	0.9
	Ptau181/ Aβ1-42	**0.06**	0.84	0.9	0.88	0.87

Adjusted Cutoffs were derived when the logistic regression model included age, sex, education, family history of AD, BMI, and MoCA score

© 2019 Garrett SL et al. *JAMA Network Open*
